# Integrative medicine for chronic pain: A cohort study using a process-outcome design in the context of a department for internal and integrative medicine

**DOI:** 10.1097/MD.0000000000004152

**Published:** 2016-07-08

**Authors:** Felix J. Saha, Alexander Brüning, Cyrus Barcelona, Arndt Büssing, Jost Langhorst, Gustav Dobos, Romy Lauche, Holger Cramer

**Affiliations:** a Department of Internal and Integrative Medicine, Kliniken Essen-Mitte, Faculty of Medicine, University of Duisburg-Essen, Essen; b Center for Integrative Medicine, Faculty of Medicine, University of Witten/Herdecke, Herdecke, Germany; c Australian Research Centre in Complementary and Integrative Medicine (ARCCIM), University of Technology Sydney, Sydney, Australia.

**Keywords:** integrative medicine, internal medicine, pain

## Abstract

**Introduction:**

Integrative medicine inpatient treatment has been shown to improve physical and mental health in patients with internal medicine conditions. The aim of this study was to investigate the effectiveness of a 2-week integrative medicine inpatient treatment in patients with chronic pain syndromes and the association of treatment success with patient-related process variables.

**Methods:**

Inpatients with chronic pain syndromes participating in a 2-week integrative medicine inpatient program were included. Patients’ pain intensity, pain disability, pain perception, quality of life, depression, and perceived stress were measured on admission, discharge, and 6 months after discharge. Likewise process variables including ability and will to change, emotional/rational disease acceptance, mindfulness, life and health satisfaction, and easiness of life were assessed.

**Results:**

A total of 310 inpatients (91% female, mean age 50.7 ± 12.4 year, 26.5% low back pain, and 22.9% fibromyalgia) were included. Using mixed linear models, significant improvements in pain intensity, pain disability, pain perception, quality of life, depression, and perceived stress were found (all *P* < 0.05). Ability to change and implementation, disease acceptance, mindfulness, life and health satisfaction, and light heartedness/easiness likewise improved (all *P* < 0.05). Improved outcomes were associated with increases in process variables, mainly ability to change and implementation, disease acceptance, life and health satisfaction, and light heartedness/easiness (*R*^2^ = 0.03–0.40).

**Conclusions:**

Results of this study suggest that a 2-week integrative medicine inpatient treatment can benefit patients with chronic pain conditions. Functional improvements are associated with improved ability to change and implementation, disease acceptance, and satisfaction.

## 1 Introduction

Integrative medicine is defined as medicine that “reaffirms the importance of the relationship between practitioner and patient, focuses on the whole person, is informed by evidence, and makes use of all appropriate therapeutic and lifestyle approaches, healthcare professionals, and disciplines to achieve optimal health and healing.”^[1]^ Integrative medicine incorporates all appropriate therapeutic approaches by all healthcare providers from both, conventional and complementary medicine, that are likely to improve an individual patient's health status.^[2]^

Chronic pain, mainly of musculoskeletal origin, is the main reason for which patients use integrative medicine approaches.^[3–5]^ The development of chronic pain is normally regarded to be caused by both, somatic and psychosocial factors^[6,7]^; thus multimodal approaches incorporating conventional somatic pain treatment as well as psychosocial, behavioral, and lifestyle-based interventions are recommended for chronic and therapy-refractory pain syndromes.^[8]^

Integrative pain treatment involves multimodal and complex interventions that strongly depend on the relationship between therapists and patients, the patients’ expectations, and motivations.^[2]^ Patients in integrative medicine settings are perceived as actively contributing to their own healing process, thus integrative medicine treatments are especially effective when the patients’ health-related cognitions and coping skills change during treatment.^[9]^ Lifestyle management, such as changing the patients’ dietary, stress management, and exercise habits, are a major part of these treatment approaches. Thus motivating the patients to adopt a healthier lifestyle during treatment and to maintaining it after discharge is an important predictor of treatment success.^[10,11]^

The Department for Internal and Integrative Medicine at Kliniken Essen-Mitte, Germany, the University of Duisburg-Essen's academic teaching hospital, was established in 1999 as a governmentally funded model institution. Developed as an integrative medicine inpatient ward from the beginning, the hospital combines conventional medicine, complementary medicine, and mind/body medicine to treat patients with chronic internal medicine diseases.^[5,10,12,13]^ Prior studies have shown positive effects of the inpatient treatment on physical and mental health in mixed patients groups.^[5]^ Chronic and therapy-refractory pain is a major reason for referral.^[5]^

The aim of this cohort study using a process-outcome design was to investigate the effects of a 2-week integrative medicine inpatient treatment at the Department for Internal and Integrative Medicine on chronic pain. A further aim was to investigate the association of treatment success with patient-related process variables, such as ability and motivation for behavioral changes, disease acceptance, and health satisfaction.

## 2 Methods

### 2.1 Design

Where applicable, this study is reported in accordance with the Strengthening the Reporting of Observational Studies in Epidemiology statement.^[14,15]^

Effects of the integrative medicine inpatient treatment were investigated in a prospective single-arm cohort study. Outcome measures were assessed in all participants at admission, at discharge, and 6 months after the end of the inpatient treatment. At 6 months, patients received the respective questionnaires and a franked envelope by mail and were asked to fill in the questionnaire and return it by mail as soon as possible. Problems or questions regarding the questionnaires were solved by phone. Outcome measures included pain intensity, pain-related disability, health-related quality of life, depression, and subjective stress. Furthermore, process variables including ability and will to change, disease acceptance, mindfulness, life and health satisfaction, and easiness of life were assessed. Associations of changes between outcome measures and process variables were analyzed. The study was approved by the Ethics Committee of the University of Duisburg-Essen (approval number: 13–5393-BO) and registered at Clinicaltrials.gov (identifier: NCT02038244) before patient recruitment.

### 2.2 Sample and setting

All patient's with an ICD-10 diagnosis of a chronic pain condition (ie, spinal pain, fibromyalgia, headache, osteoarthritis, arthritis, or other chronic pain conditions) who were referred to inpatient treatment at the Department of Internal and Integrative Medicine, Faculty of Medicine, University of Duisburg-Essen, Germany between January 2013 and July 2014 were invited to participate in this study. Written informed consent was obtained. The Department was established as a model clinic in 1999 to treat patients with chronic diseases, including those with chronic pain syndromes. Referrals come from specialist and general practitioners, with treatment costs being met by statutory health insurance and many private health insurance companies.

### 2.3 Intervention

Patients received 2 weeks of integrative inpatient hospital treatment; following individual treatment plans developed from extensive anamneses by physicians, nurses, and mind/body therapists. Treatments included conventional diagnostic and interventional medical approaches, including physiotherapy, and the use of complementary techniques. The latter included the use of traditional medicine (Traditional Chinese Medicine, acupuncture, cupping, leeching, etc) and classical naturopathy (hydrotherapy, thermotherapy, manual therapy, massage, phytotherapy, exercise, nutritional therapy, and fasting).^[5]^ Patients also received several mind/body therapy sessions, focusing on exercise, stress reduction, diet, and self-help, to empower them to adopt healthy lifestyles. These sessions were based on Harvard Medical School's Benson-Henry Institute for Mind/Body Medicine Program^[16]^ and the University of Massachusetts’ Mindfulness-Based Stress Reduction Program.^[17,18]^ Elements of cognitive restructuring were also added in this study.^[19,20]^

### 2.4 Outcome measures

*Visual Analog Scales (VAS).* Current pain, mean pain intensity and most severe pain intensity during the past 4 weeks were measured on 100-mm VAS ranging from 0 (no pain at all) to 100 (worst pain imaginable).^[21]^

*Pain Perception Scale (PPS).* The PPS measures subjectively felt pain on 2 scales: affective pain and sensory pain by 24 items.^[22]^

*Pain Disability Index (PDI).* The PDI assesses in how far specific aspects of a person's life are disrupted by chronic pain by.^[23]^

*Short Form (36) Health Survey (SF-36).* Patients’ health-related quality of life was assessed using the 36-item short form of the health survey questionnaire (SF-36).^[24]^ This tool measures an individual's quality of life on 8 dimensions and 2 main component scales (physical and mental). It has proven validity and reliability.^[24]^ Each scale ranges from 0 to 100, with higher scores indicating higher quality of life.

*Beck Depression Inventory (BDI).* Depression was assessed by the 21-item BDI.^[25]^

*Perceived Stress Scale (PSS).* Self-perceived stress level in specific situations during the last month was assessed on the 10-item German version of the PSS.^[26]^

### 2.5 Process variables

*Ability and will to Change Questionnaire*. This new instrument addresses a person's ability to perceive that changing certain aspects in life might be beneficial to get better with the health situation; their intention to change aspects in their life and behavior; whether or not they have already begun to change life and behavior on the one hand; and the successfully developed strategies how to better deal with health problems. Factor analysis pointed to 2 factors, that is, “Perception and Intention to Change” (5 items; Cronbach's alpha = .75) and “Ability to Change and Implementation (4 items; Cronbach's alpha = .77; manuscript in preparation).

*Emotional/Rational Disease Acceptance (ERDA).* The ERDA measures emotional and rational acceptance of a disease on 3 emotional scales (Positive Life Construction, Contentedness, Well Being; Rejection/Irrational Dealing with Illness; and Rejection of Guilt/Failure), and 2 rational scales (Rational Disease Acceptance and Understanding the Causes of Disease).^[27]^

*Conscious Presence and Self Control (CPSC).* The CPSC is a modified short form of the Freiburg Mindfulness Inventory, measuring mindfulness or situational awareness by 10 items on a 4-point Likert scale ranging from 0 (rarely) to 3 (almost always).^[28]^

*Brief Multidimensional Life Satisfaction Scale (BMLSS).* The BMLSS measures life satisfaction in four domains: intrinsic (myself, overall life), social (friendships, family life), external (work, where I live), and perspective (financial situation, future prospects). Two additional items assess health-related satisfaction.^[29]^

*Lightheartedness/easiness*. Several patients with chronic diseases experience an affected functional, emotional, and social well being, which may result in a self-protective “emotional withdrawal.” The intention was to make measurable distinct (emotional and behavioral) attitudes associated with a revival of vitality and zest of life, that is, positive internal attitudes, such as “light heartedness/easiness” and subsequent “social interest/contact” to external contacts. These attitudes are seen in the context of an increasing positive health/well being. The 9-item instrument differentiates two factors, light heartedness/easiness (5 items; alpha = .77), and social interest/contact (4 items; alpha = 0.79). The scale light heartedness/easiness was strongly associated with positive mood (*r* = .61), satisfaction with daily life management (*r* = 0.53), satisfaction with health situation (*r* = 0.50), with the mental component of SF-12's health-related quality of life (*r* = 0.50), and moderately with general life satisfaction (*r* = 0.47).^[30,31]^

### 2.6 Statistical analysis

Statistical analysis was based on mixed linear models using IBM SPSS software (release 20.0, IBM, Amonk, NY, USA). Values on the respective variable were regressed to the categorical covariate “time” (at admission, at discharge and at 6-month follow-up), that is, changes were analyzed across the three time points. To analyze the associations of outcome variables and process variables, linear forward stepwise regression analyses with linear outcome and linear and dichotomous predictors were conducted for all outcomes that significantly improved across time. Process variables were entered as predictors only if they had changed significantly across time. Changes from before to after the intervention and from before intervention to 6-month follow-up were used as outcome variables. Given that the influence of changes in process variables during the inpatient treatment was to be assessed, changes from before to after the intervention were used as predictor variables. To control for possible effects of clinical and sociodemographic variables, disease duration, age, and gender were additionally included in regression analyses.

*P* values <0.05 were regarded as statistically significant for all analyses. Missing data were replaced according to the manuals of the respective questionnaires. Where this was not possible, the respective questionnaire was not analyzed for this patient.

## 3 Results

### 3.1 Participants

A total of 310 patients were included of which 282 (91.0%) were female. Age ranged from 19 to 75 years with a mean age of 50.7 ± 12.4 years. The most common pain conditions included headache, rheumatic pain, and spinal pain; mean pain duration was 11.0 ± 10.7 years (Table 1). A total of 38 patients (12.3%) decided to quit the inpatient treatment earlier than the planned 2 weeks and/or withdraw their consent for study participation. From discharge to 6-month follow-up, a further 82 patients (26.5%) were lost to follow-up because they were no longer interested or did not respond.

**Table 1:**
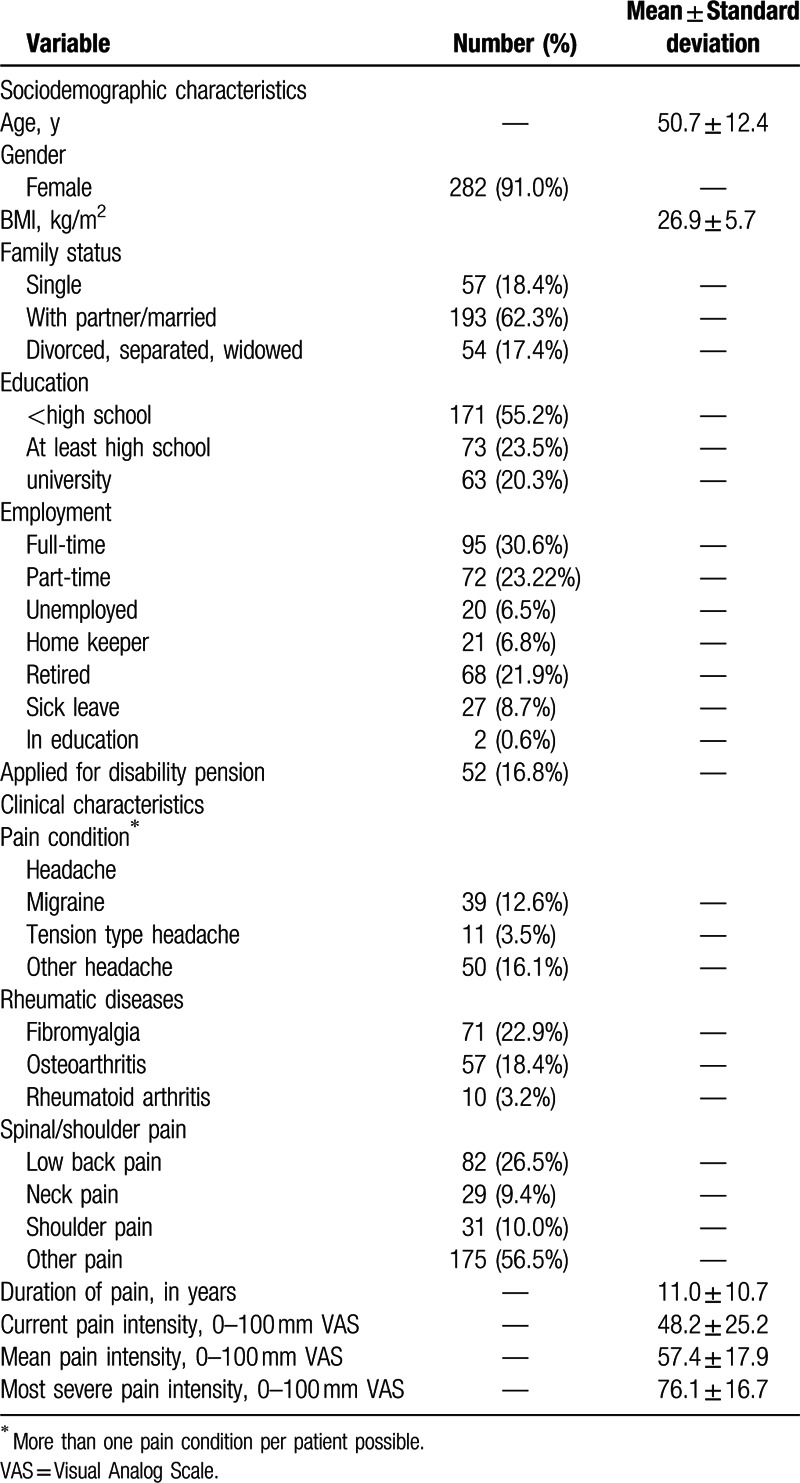
Sociodemographic and clinical characteristics at admission (n = 310).

### 3.2 Outcome measures and process variables

**Figure 1:**
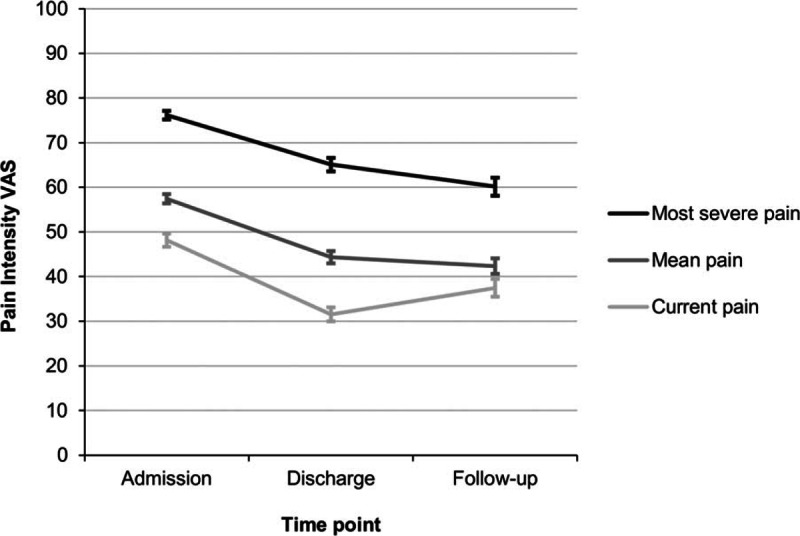
Changes in pain intensity (mean ± standard error of the mean) from admission, to discharge, and to 6 months follow-up. All *P* < 0.001. VAS = visual analog scale.

All pain VAS scores (Fig. 1), affective pain, and sensory pain domains on the PPS, depression and subjective stress decreased significantly across the course of the study, that is, from admission, to discharge, and to 6-month follow-up (Table 2). Likewise, all domains of health-related quality of life in the SF-36, namely, physical functioning, physical role functioning, bodily pain, general health, vitality, social functioning, emotional role functioning, and mental health as well as the physical and mental component scores increased significantly across the course of the study (Table 2).

**Table 2:**
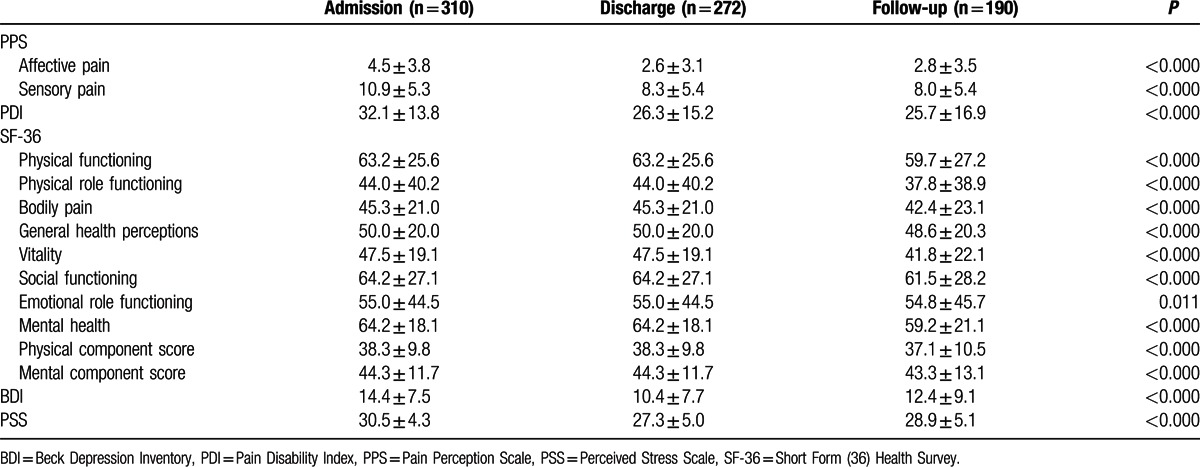
Outcome measures (mean ± standard deviation) at admission, discharge, and 6-month follow-up.

Regarding process variables, significant increases were found for ability to change and implementation, whereas perception and intention to change did not increase; all domains of emotional and rational disease acceptance; mindfulness; life satisfaction and health satisfaction; and the light heartedness/easiness domain of easiness of life, whereas social interest/contact did not change (Table 3).

**Table 3:**
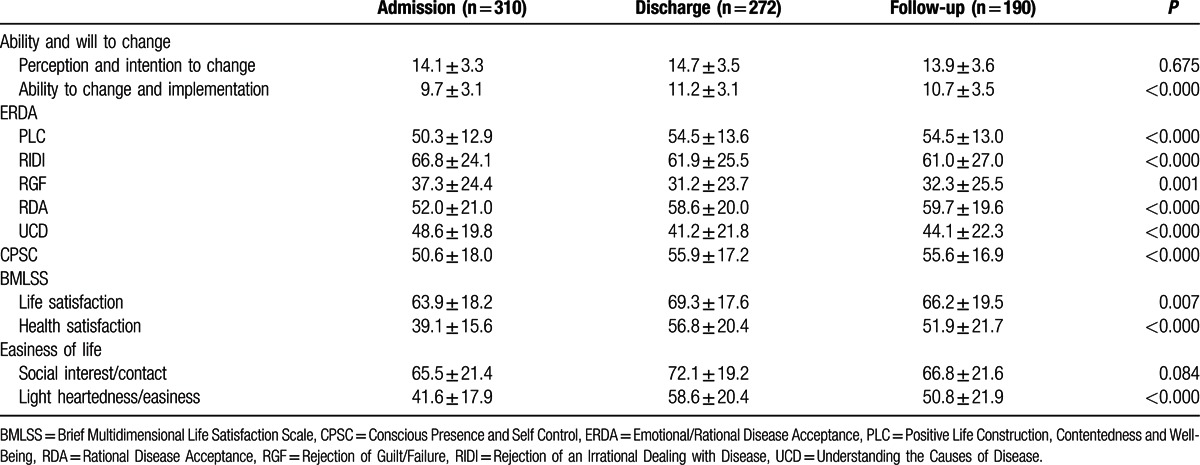
Process variables (mean ± standard deviation) at admission, discharge, and 6-month follow-up.

### 3.3 Associations of outcome measures and process variables

Regression analyses revealed significant associations with process variables for all outcomes (Tables 4 and 5). Changes from admission to discharge were mainly associated with increased health satisfaction, increased light heartedness/easiness, and reduced rejection/irrational dealing with illness (Table 4). Sustained changes in outcomes at follow-up were not only associated with increased health satisfaction, life satisfaction, and light heartedness/easiness at discharge, but also with increased ability to change and implementation, and reduced rejection of guilt/failure and rejection/irrational dealing with illness (Table 5). Other process variables were less consistently associated with changes in outcome variables. Changes in process variables explained between 3% of the variance in longer-term changes in affective and sensory pain and 40% of the variance in short-term changes in vitality (Table 4).

**Table 4:**
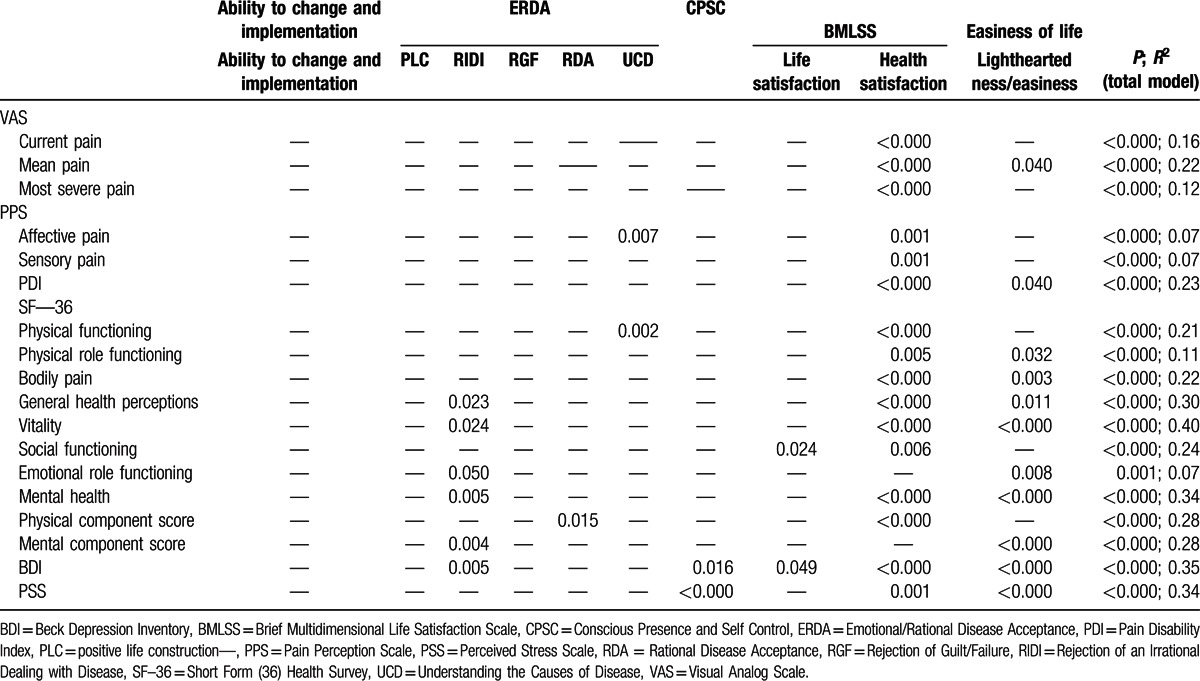
Linear multiple regression analysis: associations of changes in outcome measures (from admission to discharge) and changes in process variables (from admission to discharge). If not otherwise denoted, *P* values are shown.

**Table 5:**
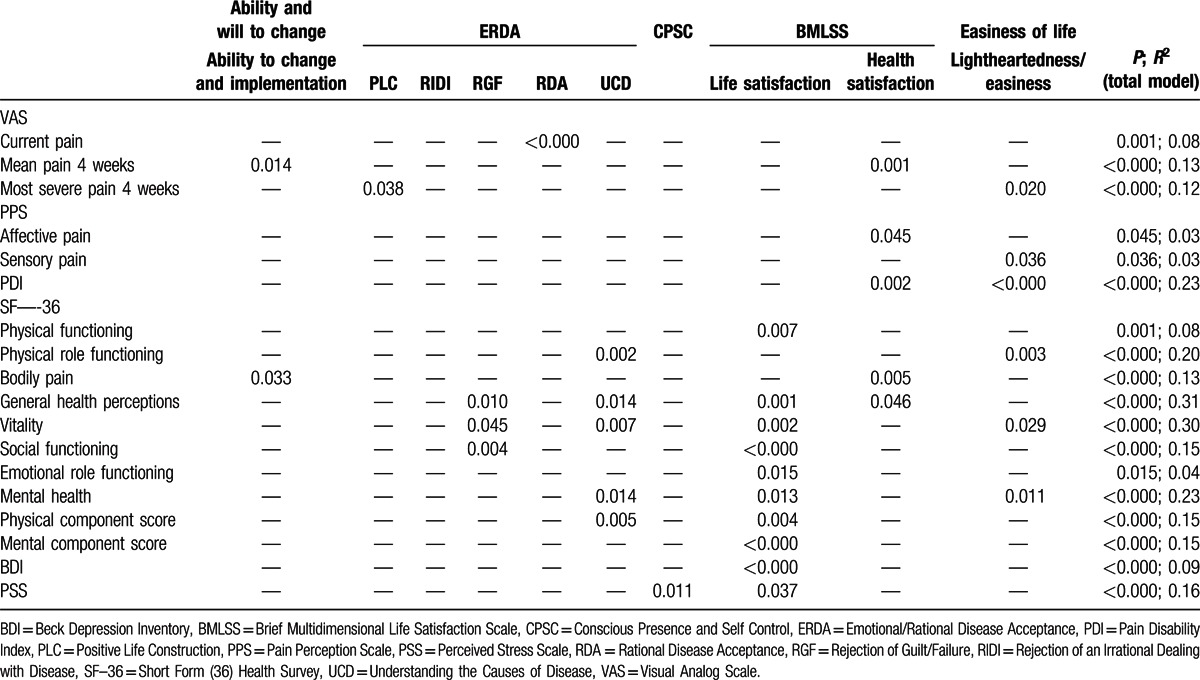
Linear multiple regression analysis: associations of changes in outcome measures (from admission to 6-month follow-up) and changes in process variables (from admission to discharge). If not otherwise denoted, *P* values are shown.

## 4 Discussion

This study investigated changes of pain, health-related quality of life, mental health, and process variables during and after a 2-week internal medicine inpatient treatment in 310 patients with chronic pain syndromes. Using mixed linear models, significant improvements in pain intensity, pain disability, all domains of quality of life, mental health, life and health satisfaction, mindfulness, disease acceptance, light heartedness/easiness, and ability to change and implementation were found. Short-term treatment success was significantly associated with increases in process variables, mainly health satisfaction and light heartedness/easiness; while ability to change and implementation, disease acceptance, and life satisfaction became more important for longer-term outcomes.

These findings are in line with prior studies on integrative medicine inpatient treatment: 2- to 3-week inpatient treatments generally increased health-related quality of life, function, and satisfaction; and decreased pain ratings, drug intake, and work absenteeism in patients with chronic pain and/or other internal medicine conditions.^[5,32–37]^ These findings were confirmed in a meta-analysis on a total of more than 7000 patients that found moderate improvements in physical and mental quality of life.^[3]^

Interestingly, while the patients’ perceived ability to change their lifestyle improved during the inpatient stay, their motivation to do so remained unchanged. Patients completing the 2-week inpatient treatment can be expected to already be relatively motivated for behavioral changes at admission. There is an about half-year long waiting period before patients can enter the inpatient treatment program and patients are required to actively participate in their treatment by attending educational exercise, stress management, dietary, and mind/body medicine sessions.^[5,9]^ Thus, patients who are not motivated to invest personal time and effort in their healing process can be expected to not start or to quit the inpatient treatment early. On the other hand, patients attending the program often do so in order to improve their coping skills, health knowledge, and ability to adopt a healthy lifestyle.^[11,12]^ Improved ability to change and implementation after the inpatient stay was not associated with treatment outcomes at discharge, but predicted lower pain at follow-up, underpinning the importance of lifestyle changes (which are mainly driven by the perceived ability to induce and maintain such changes) for long-term outcomes.^[10,11]^ Short-term effects seem to be mainly driven by practitioner-based intervention and/or externally motivated health behavior during the inpatient stay while after discharge, the patients’ intrinsic motivation and self-efficacy expectations become more important.^[10]^ This is in line with prior studies demonstrating that initiating health behavior changes depends on both, motivation for change and the perceived ability to be able to initiate and maintain such changes without extrinsic motivational factors.^[38,39]^

Disease acceptance and health satisfaction were further important predictors of short-term and long-term treatment success. This is in line with prior studies showing that pain acceptance, that is, the engagement in keeping up everyday activities and function despite the pain, is associated with less pain intensity, pain disability, and depression in patients with chronic pain syndromes.^[40,41]^ Satisfaction with health even if pain was not completely dissolved can be regarded as a consequence of increased disease acceptance.^[42]^ Specifically mind/body medical interventions such as meditation or yoga have been shown to increase pain acceptance and satisfaction in patients with chronic pain; and both concepts have been proposed as important mechanisms for these interventions’ pain relieving effects.^[43–45]^ Likewise, life and health satisfaction were important predictors of the effectiveness of an integrative medicine outpatient program for cancer patients that was conducted at the same department as the current study and build up on the same theoretical and practical foundations.^[9]^

Limitations of the study include its observational design lacking a control group. The reported effects might therefore be an overestimation due to unspecific effects and/or regression to the mean. Causal attributions can only be made for the regression analyses at follow-up where changes from admission to discharge were used as predictors. A further limitation is the multimodal approach inherent to the investigation of integrative medicine and other whole medical systems,^[46]^ making it impossible to dismantle the effects of single interventions. Given that participation in the study was not mandatory for inpatients, selection bias by only including patients with at least minimal motivation for participating in the study and treatments cannot be ruled out. Finally, in line with prior studies on integrative medicine inpatient treatment,^[5,32,34]^ drop-out rates were high at 6-month follow-up, limiting the expressiveness of the long-term findings.

In conclusion, a 2-week integrative medicine inpatient treatment can improve pain intensity and pain disability, health-related quality of life, and mental health in patients with chronic pain syndromes. These improvements seem to at least partly depend on patient-reported process variables including developing the ability for health behavior change, pain acceptance, and health satisfaction. Conclusions on the effectiveness of the program remain preliminary until comparative effectiveness and cost-effectiveness are adequately investigated.
